# *SCUBE3* Is Likely a Susceptibility Gene for Systemic Lupus Erythematosus for Chinese Populations

**DOI:** 10.1155/2020/8897936

**Published:** 2020-11-14

**Authors:** Yuan-yuan Qi, Ya-fei Zhao, Ya-ling Zhai, Xiao-xue Zhang, Xiao-yang Wang, Xina-ran Liu, Yan Cui, Xiang-hui Ning, Zhan-Zheng Zhao

**Affiliations:** ^1^Nephrology Hospital, The First Affiliated Hospital of Zhengzhou University, Henan 4500052, China; ^2^Institute of Nephrology, Zhengzhou University, Henan 4500052, China; ^3^Department of Urology, The First Affiliated Hospital of Zhengzhou University, Henan 4500052, China

## Abstract

**Background:**

Systemic lupus erythematosus (SLE) is a complex autoimmune disease with strong genetic disposition with more than 100 susceptibility genes identified until now. However, our knowledge on SLE genetic background is still limited. The present study was aimed at evaluating the role of single nucleotide polymorphisms (SNPs) in *SCUBE3*, a TGF-*β* signaling activator, with SLE susceptibility in Chinese populations.

**Methods:**

A total of 2801 individuals (490 cases and 493 controls from GWAS cohort and 1003 cases and 815 controls from our cohort) were enrolled, and SNPs located 10 kb up- and downstream of *SCUBE3* (chr6:35182190-35218609) were included in the genetic association study. Multiple layers of bioinformatics were conducted, and the levels of *SCUBE3* expression were confirmed.

**Results:**

Of the 31 SNPs in *SCUBE3* tested, 24 SNPs were significantly associated with SLE at *p* ≤ 0.05. The top locus was rs1888822 with *p* = 8.74∗10^−6^ in the discovery cohort and was confirmed by the replication cohort with *p* = 0.012. Additionally, the levels of *SCUBE3* mRNA expression were significantly lower in patients with SLE comparing with healthy controls (*p* = 4.28∗10^−4^). Further expression data from ArrayExpress showed that the expression of *SCUBE3* was also lower in CD3^+^ T cells and B cells from patients with SLE.

**Conclusions:**

Our research revealed that variants in *SCUBE3*, which encode SCUBE3 as a TGF-*β* signaling activator, can be considered as a new genetic susceptibility factor for systemic lupus erythematosus. And the reduced mRNA expression of *SCUBE3* was first reported in patients with SLE.

## 1. Introduction

Systemic lupus erythematosus (SLE) is a complex autoimmune disorder that is characterized by autoantibodies with immune complex deposition leading to multiorgan injury. The pathogenesis of SLE is multifactorial and remains unclear. Extensive research has shown that genetic factor contributes to the pathogenesis of SLE.

*SCUBE3* encodes signal peptide-CUB-EGF-like domain-containing protein 3 which is a secreted glycoprotein and is expressed during embryonic development in several tissues [1]. *SCUBE3* is dispensable for embryonic survival in the mouse [1]. *SCUBE3*^N294K/N294K^ mutants showed morphological abnormalities of the skeleton, alterations of parameters relevant for bone metabolism, changes in renal function, and hearing impairments [2]. The purified SCUBE3 protein can bound to transforming growth factor-*β* (TGF-*β*) type II receptor through the C-terminal CUB domain promoting the activation of TGF-*β* signaling [3]. It had been well documented that TGF-*β* plays a protective role in the pathogenesis of SLE. The MRL/lpr murine model of SLE benefits from intramuscular injections of cDNA expression vectors encoding for TGF-*β* with a prolonged survival [4]. SCUBE3 was associated with autoimmune diseases such as psoriasis and rheumatoid arthritis [5, 6]. Thus, the demonstration of *SCUBE3* has garnered our research interest in its possible roles in SLE.

Although the study has unveiled the genetic association of *SCUBE3* with serum Vit D levels in Crohn's Disease (CD) patients [7], the association between variants in the *SCUBE3* gene and SLE susceptibility has not yet been elucidated. Therefore, the present study was aimed at evaluating the role of single nucleotide polymorphisms (SNPs) in *SCUBE3* with SLE susceptibility in Chinese populations.

## 2. Materials and Methods

### 2.1. Study Population

The study sample comprised two cohorts (the discovery cohort 490 SLE cases vs. 493 healthy controls and replication cohort 1003 SLE cases and 815 healthy controls) comprising 2801 individuals. The discovery cohort was derived from a previously published GWAS cohort from Beijing, north of China [8], and the replication cohort was recruited from the First Affiliated Hospital of Zhengzhou University, middle east of China. All patients were diagnosed based on the 1997 American College of Rheumatology (ACR) revised criteria for SLE. The Ethical Committee of the Medical Ethics Committee of Zhengzhou University First Hospital (2019-KY-134) approved this study.

### 2.2. SNP Selection and Genotyping

We used UCSC Genome Browser 37 (GRCh37/hg19) to acquire the detailed genetic and location information of *SCUBE3*. SNPs located 10 kb up- and downstream of *SCUBE3* (chr6:35182190-35218609) were included for analysis. 31 SNPs were successfully genotyped by the ImmunoChip array by previous GWAS data (Supplementary Table 1), and the replication cohort was genotyped by Sequenom MassARRAY. The genotyping yield for the replication cohort was over 99% for both SLE cases and healthy controls.

### 2.3. Bioinformatics

The functional annotations of rs1888822 were predicted using the online bioinformatics tools and databases including rSNPBase (http://rsnp.psych.ac.cn/) [9] and RegulomeDB (http://regulome.stanford.edu/) [10]. The QTL analysis was carried out with the GTEx database (https://www.gtexportal.org/home/) [11]. The expression data of *SCUBE3* are available at ArrayExpress under accession numbers E-GEOD-13887 [12] and E-GEOD-4588.

### 2.4. The Expression of *SCUBE3* Detection

Whole blood was kept in Trizol (Life Technologies) immediately after collection and stored at -80°C for the detection of gene expression. RNA-seq of systemic lupus erythematosus (SLE) whole blood and healthy controls were conducted to determine the levels of *SCUBE3* expression.

### 2.5. Statistical Analysis

A chi-square test was carried out to compare genotype and allele frequencies of rs1888822 between cases and controls. The clinical manifestations in relation to rs1888822 genotypes were described as mean ± standarddeviation or median with range. An independent *t*-test was performed to test for the differences of *SCUBE3* expressions. Statistical analysis was performed using the SPSS 13.0 software (SPSS Inc., Chicago, IL, United States). Values were considered significant at *p* < 0.05.

## 3. Results

### 3.1. Association of *SCUBE3* Gene Polymorphisms with Susceptibility to SLE

Of the 31 SNPs tested, 24 SNPs were significantly associated with SLE at *p* ≤ 0.05 (Supplementary Table 1) [8]. Further, we identified that the most significant susceptibility locus was rs1888822 in the discovery cohort (*p* = 8.74∗10^−6^, OR 1.54, 95% CI 1.27-1.87) and confirmed the finding in the independent replication cohort (*p* = 0.012, OR 1.202, 95% CI 1.041-1.387) (Table 1). The distributions of genotypes and allele frequencies of *SCUBE3* rs1888822 among SLE patients and healthy controls are presented and analyzed in Tables 1 and 2.

### 3.2. Association of *SCUBE3* rs1888822 with Clinical Manifestations in SLE

To unveil the possible genetic associations in *SCUBE3* rs1888822 with SLE clinical manifestations, a case-only analysis was applied in the replication cohort (Table 3). The presence of malar rash, discoid rash, photosensitivity, oral ulcers, arthritis, serositis, leukopenia, lymphopenia, thrombocytopenia, anti-dsDNA antibody, and anti-Sm antibody was higher in patients with risk T allele. Importantly, SLE patients carrying risk T allele showed significantly higher levels of serum creatinine (Scr) (GT+TT vs. GG, *p* = 0.017). However, after the Bonferroni correction, there was no significant association between *SCUBE3* rs1888822 G/T polymorphism and SLE phenotype.

### 3.3. Functional Annotations of rs1888822

**Figure 1:**
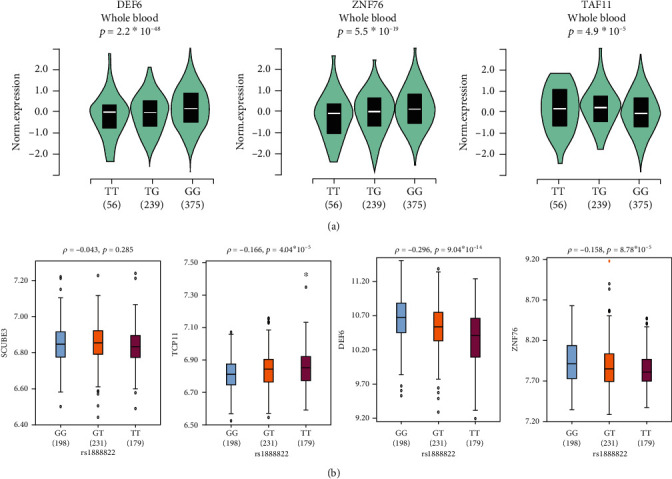
The expression of *SCUBE3* in rs188822 genotypes. Data from GTEx study (a) and the integrated analysis of the expression and genotyping data from the HapMap 3 project (b) showed the expression of *SCUBE3* in different rs188822 genotypes.

rs1888822 is annotated as a regulatory variant by rSNPBase and rank 4 (4 indicating TF binding+DNase peak) in the RegulomeDB. Further analysis by the GTEx study revealed that rs1888822 was predicted to be a potential eQTL locus associated with the expression of *SCUBE3*, *DEF6*, *ZNF76*, *TCP11*, *RPL10A*, *PPARD*, and *TAF11* in multiple tissues (Supplementary Table 2). In the whole blood, individuals carrying the risk rs1888822 allele were associated with lower expression of *DEF6* and *ZNF76* expression (Figure 1(a)). The result was also confirmed by the integrated analysis of the expression and genotyping data from the HapMap 3 project (Figure 1(b)).

### 3.4. Low Expression of *SCUBE3* in SLE Patients

**Figure 2:**
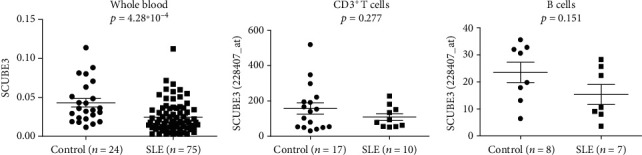
Low expression of *SCUBE3* in SLE patients. The levels of *SCUBE3* mRNA expression in whole blood from our lab and in CD3 T cells (E-GEOD-13887) and B cells (E-GEOD-4588) from the ArrayExpress database.

In order to explore the possible role of *SCUBE3* in SLE, we further examined the mRNA expression of *SCUBE3* in 75 SLE patients and 24 healthy controls. And the levels of *SCUBE3* mRNA expression were significantly lower in patients with SLE comparing with healthy controls (*p* = 4.28∗10^−4^) (Figure 2(a)). Moreover, we searched public gene expression databases, ArrayExpress, consisting of a large number of whole blood gene expression profiles. We adopted the expression data from CD3 T cells (E-GEOD-13887) and B cells (E-GEOD-4588) from peripheral blood. Comparing with healthy donors, the expression of *SCUBE3* was also lower in CD3 T cells (*p* = 0.277) and B cells (*p* = 0.151) from patients with SLE (Figures 2(b) and 2(c)).

## 4. Discussion

In recent years, there have been numerous genetic association studies on a variety of SLE susceptibility. However, these genes can only explain a small portion of the genetic liability for SLE, as SLE is widely known to be a polygenic disorder with many risk genes of small effects. In this study, we examined the impact of polymorphisms within the *SCUBE3* gene and SLE susceptibility.

In the present study, a genetic discovery-replication study was performed and the association between rs1888822 *SCUBE3* and SLE susceptibility was identified in the Chinese population. In the Korean population, the genetic association result between rs1888822 *SCUBE3* and SLE susceptibility was 9.88∗10^−4^ (OR 1.17, 95% CI 1.06-1.28) [8]. Our research exposed that patients carrying risk T allele (GT+TT genotypes) may have an impact on elevated Scr value which is one of the important biomarkers to assess renal function. Considering the potential vulnerable renal function, SLE patients with risk genotype of rs1888822 *SCUBE3* were suggested to monitor serum creatinine and urinalysis more actively. Whether the expression of rs1888822 *SCUBE3* was affected by the disease conditions such as high creatinine/renal failure was interesting and well worth to be investigated in separated projects in the future. Despite the correlation between the level of Scr and rs1888822 genotypes, we failed to identify more clues to connect rs1888822 genotypes and clinical manifestations. Replication studies from different populations are required, a large sample size of SLE cases is crucial to confirm our genetic finding, and more clinical connections can be expected to be discovered.

SLE is an autoimmune inflammatory disease in which the abnormalities of various pro- and anti-inflammatory cytokines played crucial pathogenic roles. TGF-*β* was mainly produced by regulatory T cells (Treg) and has both immunoregulatory and proinflammatory properties [13, 14]. The levels of TGF-*β* were significantly lower in patients with SLE and were negatively correlated with disease activity [15–18]. Disruption of the TGF-*β*1 gene in mice resulted in immune and inflammatory disorders resembling the symptom of SLE, including the formation of autoantibodies and renal impairment [19–22]. Treatment with ATG+latent TGF-beta1 synergistically inhibited the progression of proteinuria and albuminuria and provided a significant improvement in long-term survival [23]. The above evidences suggested that the protective role of TGF-*β*1 had been well recognized in the pathogenesis of SLE [24]. The C-terminal CUB domain of SCUBE3 protein can activate TGF-*β* signaling by bounding to the TGF-*β* type II receptor [3]. Both data from our lab in whole blood and data from the ArrayExpress database in CD3 T cells (E-GEOD-13887) and B cells (E-GEOD-4588) confirmed the decreased mRNA expression of *SCUBE3*. Thus, we speculated that the reduced expression of *SCUBE3* might contribute to the insufficient activation of the TGF-*β* signaling pathway promoting the development of SLE. Because our knowledge of SCUBE3 biological function in autoimmune disease remains limited, future biological studies on lupus-prone mice might provide more detailed information and carry out essential clues for SCUBE3 in the pathogenesis of SLE. The function of TGF-*β*1 is inherently a double-edged sword by acting as both immune suppressor and immune reaction promoter [25, 26]. As a TGF-*β* signaling activator, additional studies are needed to evaluate the value of targeting SCUBE3 for autoimmune disease therapy.

Variants in *DEF6* had been confirmed associated with SLE susceptibility with robust evidences [8]. Notably, the concomitant lack of DEF6 and Swap-70 in C57BL/6 mice spontaneously develop a lupus-like syndrome in aging female mice [27]. ZNF76, which functions as a transcriptional repressor, had a strong inhibitory effect on p53 in various cell lines [28]. Bioinformatics analysis indicated rs1888822 was a functional locus, particularly the eQTL effects. Individuals carrying rs1888822 risk T allele were associated with lower expression of *DEF6* and *ZNF76*. Whether rs1888822 promotes the pathogenesis of SLE through the reduced expression of *DEF6* or *ZNF76* requires further investigation.

## 5. Conclusions

Our research revealed that variants in *SCUBE3* can be considered as a new genetic susceptibility factor for systemic lupus erythematosus. Moreover, rs1888822 was a potential functional locus which might promote the pathogenesis of SLE by eQTL effects. Additionally, the reduced mRNA expression of *SCUBE3* was first reported in SLE patients.

## Figures and Tables

**Table 1:** Association of rs1888822 in the *SCUBE3* gene with susceptibility to systemic lupus erythematosus.

Chr.	Gene	SNP	Position (hg19)	Minor allele	Discovery stage (490/493)			Replication stage (1003/815)			Meta-analysis	
					MAF (case/control %)	*p* value	OR (95% CI)	MAF (case/control %)	*p* value	OR (95% CI)	*p* value	OR (95% CI)
6	*SCUBE3*	rs1888822	35183149	T	36.7/28.6	8.74∗10^−6^	1.54 (1.27-1.87)	32.0/28.1	0.012	1.202 (1.041-1.387)	4.14∗10^−6^	1.31 (1.17-1.47)

**Table 2:** Genotype frequency of *SCUBE3* rs1888822 in SLE patients and healthy controls.

rs1888822	Genotype	SLE *N* (%)	Controls *N* (%)	OR (95% CI)	*p* value
Codominant	GG	461 (46.3)	412 (50.9)	Reference	
	GT	431 (43.3)	340 (42.0)	1.133 (0.933-1.376)	0.209
	TT	103 (10.4)	58 (7.2)	1.587 (1.121-2.248)	8.92∗10^−3^
Additive model	GG	461 (46.3)	412 (50.9)	Reference	
	TT	103 (10.4)	58 (7.2)	1.587 (1.121-2.248)	8.92∗10^−3^

**Table 3:** Correlation between rs1888822 and clinical relevance.

Clinical manifestations		GG (*n* = 461)	GT+TT (*n* = 534)	*p* value
Gender (male, %)		27 (5.9)	44 (8.2)	0.145
Onset age (mean ± SD)		31 ± 13	30 ± 13	0.349
Malar rash (+, %)		113 (24.5)	138 (25.8)	0.630
Discoid rash (+, %)		2 (0.4)	5 (0.9)	0.344
Photosensitivity (+, %)		15 (3.3)	27 (5.1)	0.159
Oral ulcers (+, %)		33 (7.2)	39 (7.3)	0.930
Nonerosive arthritis (+, %)		125 (27.1)	152 (28.5)	0.636
Pleuritis or pericarditis (+, %)		31 (6.7)	51 (9.6)	0.141
Renal disorder	Scr (median QR)	54 (47-65)	56 (48-70)	0.017
	24 h UTP (mean ± SD)	2.07 ± 2.73	2.80 ± 9.26	0.316
	Pathological classifications (I+II/III+IV/V, %)	6 (6.8)/71 (80.7)/11 (12.5)	13 (11.6)/86 (76.8)/13 (11.6)	0.517
Neurologic disorder (+, %)		19 (4.1)	16 (3.0)	0.337
Hematologic disorder	Hemolytic anemia (+, %)	9 (2.0)	9 (1.7)	0.753
	Leukopenia (+, %)	107 (23.9)	138 (26.2)	0.409
	Lymphopenia (+, %)	181 (40.7)	220 (42.0)	0.680
	Thrombocytopenia (+, %)	109 (24.4)	134 (25.5)	0.695
Immunologic disorder	Anti-dsDNA (+, %)	257 (62.1)	310 (63.9)	0.569
	Anti-Sm (+, %)	53 (15.5)	78 (19.0)	0.211
	C3 (mean ± SD)	0.73 ± 0.38	0.71 ± 0.34	0.269
	C4 (mean ± SD)	0.14 ± 0.12	0.14 ± 0.14	0.711
SLEDAI (mean ± SD)		4.6 ± 4.2	4.7 ± 4.1	0.828
Treatments	Glucocorticoids (+, %)	300 (65.1)	351 (65.7)	0.841
	Immunosuppressants (+, %)	105 (22.8)	111 (20.8)	0.448

## Data Availability

The data that support the findings of this study are available from the corresponding author upon reasonable request.

## Abbreviations

SLE: Systemic lupus erythematosus
LN: Lupus nephritis
GWAS: Genome-wide association study
eQTL: Expression quantitative trait loci
SNP: Single nucleotide polymorphism
SCUBE3: Signal peptide-CUB-EGF-like domain-containing protein 3
ZNF76: Zinc finger protein 76
DEF6: Differentially expressed in FDCP 6 homolog.

## Supplementary Materials

Supplementary materials Supplementary Table 1. Association results of SNPs in *SCUBE3* and SLE susceptibility. Supplementary Table 2. eQTLs of rs1888822.
