# Supplementary material for: SCUBE3 Is Likely a Susceptibility Gene for Systemic Lupus Erythematosus for Chinese Populations
Source: J Immunol Res. 2020 Nov 14;2020:8897936. doi: 10.1155/2020/8897936 (PMC7683159; doi:10.1155/2020/8897936)
Supplement: Supplementary materials — Supplementary Table 1. Association results of SNPs in SCUBE3 and SLE susceptibility. Supplementary Table 2. eQTLs of rs1888822. [file 8897936.f1.docx]

***SCUBE3* is likely a susceptibility gene for systemic lupus erythematosus for Chinese populations**

Yuan-yuan Qi^12#^, Ya-fei Zhao^12#^, Ya-ling Zhai^12^, Xiao-xue Zhang^12^, Xiao-yang Wang^12^, Xina-ran Liu^12^, Yan Cui^12^, Xiang-hui Ning^3^, Zhan-Zheng Zhao^12*^

**AUTHORS’ INSTITUTION AND AFFILIATION**

1.Nephrology Hospital, the First Affiliated Hospital of Zhengzhou University, Henan 4500052, China;

2. Institute of Nephrology, Zhengzhou University, Henan 4500052, China;

3. Department of Urology, the First Affiliated Hospital of Zhengzhou University, Henan 4500052, China.

^#^: These authors contribute equally to this work.

CORRESPONDING AUTHOR

Dr. Zhan-zheng Zhao, MD & PhD;

Email: zhanzhengzhao@zzu.edu.cn

Nephrology Hospital, the First Affiliated Hospital of Zhengzhou University,

Institute of Nephrology, Zhengzhou University

No.1, Jianshe Road, Erqi District

Zhengzhou 4500052, P.R China

| Supplementary table1. Association results of SNPs in *SCUBE3* and SLE susceptibility. | | | | | | |
| --- | --- | --- | --- | --- | --- | --- |
| SNPs | Chr | Pos (hg19) | Minor allele | MAF (Case/Control, %) | P-value | OR(95%CI) |
| rs1108709 | 6 | 35172194 | C | 40/31.3 | 6.10*10^-5^ | 1.46(1.21-1.76) |
| rs3734252 | 6 | 35173757 | A | 6.5/4.3 | 2.58*10^-2^ | 1.57(1.05-2.34) |
| rs6928769 | 6 | 35174858 | T | 38.5/29.4 | 2.05*10^-5^ | 1.5(1.25-1.81) |
| rs3800393 | 6 | 35179541 | C | 40/31.3 | 6.10*10^-5^ | 1.46(1.21-1.76) |
| rs3800392 | 6 | 35179624 | T | 38.5/29.3 | 1.79*10^-5^ | 1.51(1.25-1.82) |
| rs3800391 | 6 | 35180781 | C | 2.1/2.3 | 7.76*10^-1^ | 0.92(0.50-1.67) |
| rs9368854 | 6 | 35181236 | T | 36.2/27.6 | 3.97*10^-5^ | 1.49(1.23-1.81) |
| rs1888822 | 6 | 35183149 | T | 36.6/27.3 | 8.74*10^-6^ | 1.54(1.27-1.87) |
| imm_6_35292689 | 6 | 35184711 | C | 36.2/27.8 | 6.09*10^-5^ | 1.48(1.22-1.79) |
| rs3800388 | 6 | 35186149 | T | 36.3/27.6 | 3.24*10^-5^ | 1.5(1.24-1.81) |
| rs3800387 | 6 | 35186501 | A | 36.3/27.7 | 4.03*10^-5^ | 1.49(1.23-1.80) |
| rs1987673 | 6 | 35186646 | G | 40/31.3 | 6.10*10^-5^ | 1.46(1.21-1.76) |
| rs115453475 | 6 | 35187227 | T | 0.7/0.7 | 9.91*10^-1^ | 1.01(0.35-2.88) |
| rs3800386 | 6 | 35187477 | T | 36.3/28 | 7.61*10^-5^ | 1.47(1.21-1.78) |
| rs9394282 | 6 | 35188925 | G | 1.7/1.5 | 7.17*10^-1^ | 1.14(0.57-2.29) |
| rs9394283 | 6 | 35189636 | A | 38.9/29.6 | 1.47*10^-5^ | 1.51(1.25-1.83) |
| rs3800385 | 6 | 35189999 | G | 39/29.6 | 1.22*10^-5^ | 1.52(1.26-1.83) |
| rs1041528 | 6 | 35190120 | T | 38.6/29.3 | 1.41*10^-5^ | 1.52(1.26-1.83) |
| rs9469964 | 6 | 35191519 | G | 39/29.6 | 1.22*10^-5^ | 1.52(1.26-1.83) |
| rs763155 | 6 | 35193773 | A | 1.9/1.5 | 4.78*10^-1^ | 1.28(0.65-2.53) |
| rs4713842 | 6 | 35194632 | G | 1.5/0.6 | 4.67*10^-2^ | 2.54(0.98-6.57) |
| rs3800383 | 6 | 35197814 | C | 36.3/27.7 | 4.03*10^-5^ | 1.49(1.23-1.80) |
| rs1929848 | 6 | 35200350 | G | 2.1/1.6 | 3.96*10^-1^ | 1.33(0.69-2.56) |
| rs1013907 | 6 | 35201858 | T | 38.5/29.3 | 1.79*10^-5^ | 1.51(1.25-1.82) |
| rs114716259 | 6 | 35202026 | A | 0.3/0.7 | 2.08*10^-1^ | 0.43(0.11-1.67) |
| rs2395614 | 6 | 35202883 | C | 40/31.2 | 4.97*10^-5^ | 1.47(1.22-1.77) |
| rs13214290 | 6 | 35203203 | T | 39/29.6 | 1.22*10^-5^ | 1.52(1.26-1.83) |
| rs4713843 | 6 | 35203745 | T | 1.5/0.6 | 4.67*10^-2^ | 2.54(0.98-6.57) |
| rs1929849 | 6 | 35205541 | T | 38.4/29.2 | 1.76*10^-5^ | 1.51(1.25-1.82) |
| rs734538 | 6 | 35205881 | G | 1.9/1.5 | 4.78*10^-1^ | 1.28(0.65-2.53) |
| rs732594 | 6 | 35206553 | A | 30.4/38.3 | 2.15*10^-4^ | 0.7(0.58-0.85) |

Supplementary Table 2. eQTLs of rs1888822.

| Gene | P-Value | NES | Tissue |
| --- | --- | --- | --- |
| *DEF6* | 2.20E-48 | 0.3 | Whole Blood |
| *ZNF76* | 5.50E-19 | 0.13 | Whole Blood |
| *DEF6* | 4.90E-14 | 0.2 | Nerve - Tibial |
| *TCP11* | 2.40E-11 | -0.44 | Adipose - Subcutaneous |
| *SCUBE3* | 5.30E-11 | 0.21 | Cells - Cultured fibroblasts |
| *TCP11* | 1.00E-10 | -0.44 | Skin - Sun Exposed (Lower leg) |
| *RPL10A* | 2.80E-10 | -0.13 | Artery - Tibial |
| *ZNF76* | 1.20E-09 | 0.26 | Testis |
| *ZNF76* | 2.00E-09 | 0.22 | Adrenal Gland |
| *RPL10A* | 2.40E-09 | -0.19 | Esophagus - Muscularis |
| *ZNF76* | 2.40E-09 | 0.15 | Muscle - Skeletal |
| *DEF6* | 3.50E-09 | 0.27 | Pituitary |
| *TCP11* | 1.20E-08 | -0.41 | Skin - Not Sun Exposed (Suprapubic) |
| *DEF6* | 3.40E-08 | 0.2 | Heart - Left Ventricle |
| *DEF6* | 6.00E-08 | 0.44 | Ovary |
| *DEF6* | 1.20E-07 | 0.13 | Lung |
| *ZNF76* | 2.80E-07 | 0.13 | Artery - Aorta |
| *ZNF76* | 3.00E-07 | 0.14 | Artery - Tibial |
| *DEF6* | 4.30E-07 | 0.25 | Esophagus - Gastroesophageal Junction |
| *ZNF76* | 4.40E-07 | 0.14 | Esophagus - Mucosa |
| *RPL10A* | 0.0000023 | -0.095 | Nerve - Tibial |
| *RPL10A* | 0.0000026 | -0.098 | Muscle - Skeletal |
| *DEF6* | 0.0000033 | 0.15 | Adipose - Visceral (Omentum) |
| *TCP11* | 0.0000035 | -0.34 | Esophagus - Mucosa |
| *RPL10A* | 0.0000036 | -0.11 | Adipose - Visceral (Omentum) |
| *RPL10A* | 0.0000037 | -0.19 | Colon - Sigmoid |
| *DEF6* | 0.0000042 | 0.27 | Cells - Cultured fibroblasts |
| *ZNF76* | 0.0000044 | 0.14 | Heart - Atrial Appendage |
| *TCP11* | 0.0000059 | -0.088 | Testis |
| *TCP11* | 0.0000076 | -0.37 | Heart - Atrial Appendage |
| *TCP11* | 0.0000084 | -0.4 | Pituitary |
| *ZNF76* | 0.0000092 | 0.15 | Adipose - Subcutaneous |
| *TCP11* | 0.000012 | -0.33 | Adipose - Visceral (Omentum) |
| *DEF6* | 0.000013 | 0.1 | Artery - Tibial |
| *ZNF76* | 0.000016 | 0.19 | Brain - Cerebellum |
| *DEF6* | 0.000017 | 0.12 | Thyroid |
| *RPL10A* | 0.000019 | -0.089 | Thyroid |
| *TCP11* | 0.000021 | -0.27 | Artery - Tibial |
| *PPARD* | 0.000024 | -0.13 | Skin - Not Sun Exposed (Suprapubic) |
| *TCP11* | 0.000035 | -0.33 | Breast - Mammary Tissue |
| *DEF6* | 0.000048 | 0.38 | Cells - EBV-transformed lymphocytes |
| *TAF11* | 0.000049 | -0.099 | Whole Blood |
| *DEF6* | 0.000055 | 0.14 | Esophagus - Muscularis |
| *DEF6* | 0.000067 | 0.092 | Adipose - Subcutaneous |
| *DEF6* | 0.000072 | 0.31 | Brain - Cerebellum |
| *DEF6* | 0.000083 | 0.18 | Brain - Caudate (basal ganglia) |
| *RPL10A* | 0.00011 | -0.11 | Artery - Aorta |
| *ZNF76* | 0.00013 | 0.14 | Colon - Transverse |
| *ZNF76* | 0.00013 | 0.088 | Esophagus - Muscularis |
| *TCP11* | 0.00014 | -0.2 | Lung |
| *RPL10A* | 0.00029 | -0.066 | Adipose - Subcutaneous |
| *DEF6* | 0.00039 | 0.14 | Muscle - Skeletal |
